# Pembrolizumab-Induced Immune-Mediated Hepatitis and Concurrent Hepatitis B Reactivation in a Patient With Non-Small Cell Lung Cancer

**DOI:** 10.7759/cureus.11522

**Published:** 2020-11-17

**Authors:** Shanker Kundumadam, Bashar Mohamad, Arun Muthusamy, Pradeep R Kathi, Murray N Ehrinpreis

**Affiliations:** 1 Internal Medicine, Wayne State University School of Medicine, Detroit, USA; 2 Gastroenterology and Hepatology, Wayne State University, Detroit, USA; 3 Gastroenterology and Hepatology, Cedar Valley Digestive Health Center, Iowa, USA; 4 Internal Medicine/Gastroenterology, University of Arizona, Tucson, USA; 5 Gastroenterology, Wayne State University, Detroit, USA

**Keywords:** hepatitis, pembrolizumab

## Abstract

New immuno-therapeutic agents like pembrolizumab used in cancer treatment are known to cause immune-mediated hepatitis. Most of these cases are straightforward when the onset of transaminitis correlates with the introduction of the medication. This agent causing hepatitis B reactivation has been reported only once. To have both these adverse effects occurring at the same time in a patient is uncommon and presents a clinical challenge.

Our patient was a 49-year-old gentleman diagnosed with metastatic adenocarcinoma of the lung seven months ago. He was started on pembrolizumab, as the malignant tissue obtained during biopsy had high program death-ligand 1 (PDL1) expression. On reviewing the labs ordered during the time of cancer diagnosis, this man has evidence of chronic hepatitis B with positive hepatitis B surface antigen and positive hepatitis B core immunoglobulin G (IgG) antibody. He presented with acute hepatitis, and workup showed features of hepatitis B reactivation, but the extent of reactivation was not adequate to explain the presentation, hence investigations were pursued. This led the way to the diagnosis of a combined hepatitis B reactivation and drug-induced immune hepatitis in this case. He responded promptly to the withdrawal of the agent and steroids. On follow-up, his liver function panel had significantly improved.

This case is very unique in two aspects. First, to our knowledge, there is only one case reported of pembrolizumab-induced hepatitis B reactivation. In addition, our patient also had immune-mediated hepatitis induced by pembrolizumab. It is very rare to have a combination of these two presentations to be seen in a patient at the same time. Pembrolizumab-induced immune hepatitis can coexist with hepatitis B reactivation following therapy with this agent.

## Introduction

New immunotherapeutic agents like pembrolizumab used in cancer treatment are known to cause immune-mediated hepatitis. Most of these cases are straightforward when the onset of transaminitis correlates with the introduction of the medication. This agent causing hepatitis B reactivation has been reported only once. To have both these adverse effects occurring at the same time in a patient is uncommon and presents as a clinical challenge.

## Case presentation

Our patient is a 49-year-old gentleman who was diagnosed with metastatic adenocarcinoma of the lung seven months back. He also had biopsy-proven metastases to bones and the adrenal gland. He was started on pembrolizumab, as the malignant tissue obtained during biopsy had high program death-ligand 1 (PDL1) expression. The medication was started four months ago. His only other active medication was an opioid for pain control. In his recent follow-up visit to the oncologist, it was found that he was having elevated liver enzymes, and he was admitted to the hospital for further evaluation. On reviewing the labs ordered during the time of cancer diagnosis, this gentleman has evidence of chronic hepatitis B with positive hepatitis B surface antigen and positive hepatitis B core immunoglobulin G (IgG) antibody (negative IgM antibody). His liver enzymes seven months ago showed an alanine transaminase (ALT) of 25 Units/L, aspartate transaminase (AST) of 22 Units/L, and alkaline phosphatase (ALP) of 207 Units/L. At the current admission, his workup revealed a considerable elevation of liver enzymes from baseline, with an ALT of 508 Units/L, AST of 627 Units/L, and ALP of 256 Units/L. Over the next few days, this would continue to trend up and reached a peak of ALT: 630 Units/L and AST: 670 Units/L. His bilirubin levels were normal and so was the hemogram. Ultrasound of the liver with Doppler was performed at this point, which showed an echogenic, mildly enlarged liver but was otherwise unremarkable. A viral hepatitis panel was ordered at this point. Interestingly, it showed a positive IgM hepatitis B core antibody. This was very suggestive of hepatitis B reactivation.

We held the pembrolizumab and started the patient on tenofovir. In the meantime, the hepatitis B viral polymerase chain reaction (PCR) was under process. The viral PCR result came back as 4450 IU/ml. Considering the extent of the transaminitis, it was ascertained that this viral load would not explain it. The focus of the case turned towards pembrolizumab-induced immune-mediated hepatitis at this point. A liver biopsy followed, which showed severe portal inflammation with interface hepatitis, which is circumferential around the portal tracts containing lymphocytes, and plasma cells. Biopsy findings favored immune-mediated hepatitis. He was started on oral prednisone 70 mg daily at this point. Over the next couple of days, the liver enzymes started trending down. At the time of discharge, the liver enzymes were as follows: ALT: 515 Units/L, AST: 435 Units/L, and ALP: 193 Units/L. On further outpatient follow-up a month later, his bilirubin and ALP had normalized, with near-normal transaminases (ALT 53 Units/L and AST 18 Units/L). The plan is to reassess him in two to four weeks with a repeat lab draw while on steroid taper.

## Discussion

Pembrolizumab is a monoclonal antibody used in the treatment of various cancers. Immunotherapy agents like pembrolizumab targeting the PD1/ PDL1 signaling pathway are being more frequently used in melanoma, non-small cell lung cancer, etc. [1]. Immune-mediated adverse effects are commonly seen with pembrolizumab and other agents in this category. They were noted in less than 2% of the patients taking this medication [2-4]. These include dermatological manifestations, hepatitis, colitis, endocrine dysfunction, and pneumonitis. The mechanism in which these agents cause immune-mediated hepatitis is not fully understood. It has been hypothesized that the break in the PD1/PDL1 signaling pathway makes the liver vulnerable to cytotoxic T lymphocytes.

This case is very unique in two aspects. First, to our knowledge, there is only one case reported of pembrolizumab-induced hepatitis B reactivation [5]. In addition, our patient also had immune-mediated hepatitis induced by pembrolizumab. It is very rare to see a combination of these two presentations in a patient at the same time. Considering the fact that immune-mediated hepatitis is more commonly seen, it is possible that in cases of hepatic dysfunction following pembrolizumab initiation, the reactivation of chronic viral hepatitis B may not be sought after. This will, in turn, lead to therapy completely focusing on immunosuppression and withholding the medication but not address the need for anti-viral therapy. This could prove detrimental later due to extensive hepatic complications that can occur from hepatitis B reactivation. Determining between hepatitis B and pembrolizumab as the culprit in these situations can be challenging, as in our case. The mismatch between the magnitude of the viral load and the enzyme elevation may give a clue.

Most cases of immune-mediated hepatitis present with an asymptomatic elevation of liver enzymes [6]. Rarely, acute liver failure occurs as a result of immune-related hepatitis [7]. Imaging studies may either reveal normal findings or show hepatomegaly, peri-portal edema, and adenopathy [8]. Histopathology in similar cases has been described in the literature. It can show either a predominantly hepatocyte injury pattern with panlobular hepatitis and prominent peri-venular infiltrate with endotheliitis or a biliary pattern with predominant injury to bile ducts. Management includes immunosuppression with corticosteroids, mycophenolate mofetil, or other agents. Tumor necrosis factor-alpha antagonists are not used for immune-mediated hepatitis but can be used for other toxicities [9]. In cases like ours, in addition to the immunosuppression, anti-viral agents to target the hepatitis B virus should be initiated simultaneously to prevent further liver damage.

## Conclusions

Pembrolizumab and other similar immunotherapy agents have the potential to cause concurrent immune-mediated hepatitis and hepatitis B reactivation, thereby producing a significant diagnostic dilemma.
